# Partnering with Health Care Systems to Assess Tobacco Treatment Practices and Beliefs Among Clinicians: Evaluating the Process

**DOI:** 10.5888/pcd11.130277

**Published:** 2014-05-29

**Authors:** Michael D. Celestin, Alton Hart, Sarah Moody-Thomas

**Affiliations:** Author Affiliations: Alton Hart Jr, Virginia Department of Health Crater Health District, Petersburg, Virginia; Sarah Moody-Thomas, Louisiana State University Health Sciences Center School of Public Health, New Orleans, Louisiana.

## Abstract

**Background:**

Tobacco is a major cause of preventable illness and death. However, clinician use of an evidence-based guideline for treatment of tobacco use is low. This case study describes the process for conducting a pre-intervention assessment of clinician practices and beliefs regarding treatment of tobacco use.

**Community Context:**

Louisiana State University Health System, one of the largest safety-net public hospital systems in the United States, consists of 10 facilities in population centers across the state of Louisiana. The system serves a large proportion of the state’s underinsured and uninsured, low-income, and racial/ethnic minority populations, groups that have high rates of tobacco use.

**Methods:**

Activities included 1) partnering with hospital administrators to generate support for conducting a clinician assessment, 2) identifying and adapting a survey tool to assess clinicians’ practices and beliefs regarding treatment of tobacco use, 3) developing a survey protocol and obtaining approval from the institutional review board, and 4) administering the survey electronically, using the hospital’s e-mail system.

**Outcome:**

Existing partnerships and system resources aided survey administration. Use of the hospital’s internal e-mail system and distribution of an online survey were effective means to engage clinicians. Following notification, 43.6% of 4,508 clinicians opened their e-mail containing the invitation letter with a Web link to the survey; of these, 83.1% (1,634) completed the survey.

**Interpretation:**

Partnering with stakeholders and using existing resources within the health care system are essential to successful implementation of a system-wide survey of clinician practices and beliefs regarding treatment of tobacco use.

## MEDSCAPE CME

Medscape, LLC is pleased to provide online continuing medical education (CME) for this journal article, allowing clinicians the opportunity to earn CME credit.

This activity has been planned and implemented in accordance with the Essential Areas and policies of the Accreditation Council for Continuing Medical Education through the joint sponsorship of Medscape, LLC and Preventing Chronic Disease. Medscape, LLC is accredited by the ACCME to provide continuing medical education for physicians.

Medscape, LLC designates this Journal-based CME activity for a maximum of 1 **AMA PRA Category 1 Credit(s)™**. Physicians should claim only the credit commensurate with the extent of their participation in the activity.

All other clinicians completing this activity will be issued a certificate of participation. To participate in this journal CME activity: (1) review the learning objectives and author disclosures; (2) study the education content; (3) take the post-test with a 70% minimum passing score and complete the evaluation at www.medscape.org/journal/pcd (4) view/print certificate.

**Release date: May 29, 2014; Expiration date: May 29, 2015**

### Learning Objectives

Upon completion of this activity, participants will be able to:

- Distinguish sociodemographic variables associated with higher rates of smoking
- Analyze physicians’ performance in the clinical management of smoking
- Evaluate a survey program of physicians and nurses regarding the clinical management of smoking

**EDITORS**

Teresa L. Ramsey, Writer-Editor, *Preventing Chronic Disease*. Disclosure: Teresa L. Ramsey has disclosed no relevant financial relationships.

**CME AUTHOR**

Charles P. Vega, MD, Associate Professor and Residency Director, Department of Family Medicine, University of California, Irvine. Disclosure: Charles P. Vega, MD, has disclosed no relevant financial relationships.

**AUTHORS AND CREDENTIALS**

Disclosures: Michael D. Celestin, Jr., MA, CHES, CTTS, Alton Hart, Jr., MD, MPH, Sarah Moody-Thomas, PhD, have disclosed no relevant financial relationships.

Affiliations: Michael D. Celestin, Jr., Sarah Moody-Thomas, Louisiana State University Health Sciences Center School of Public Health, New Orleans, Louisiana; Alton Hart, Jr., Virginia Department of Health Crater Health District, Petersburg, Virginia.

## Background

Tobacco use is the foremost cause of preventable illness and death in the United States (1). Smoking causes coronary heart disease and lung cancer and is associated with many other deleterious health effects (2). Although prevalence of tobacco use in the United States has modestly decreased during the past 7 years, prevalence in Louisiana (22%) has not changed (3,4). Louisiana residents with no insurance (34%), low income (32%), and less than a high school education (36%), and who are men (25%) are more likely to report tobacco use (4) and to receive health care through public and safety-net health systems. These facts highlight the need for greater use of evidence-based interventions and strategies for addressing tobacco use in disparate populations.

More must be done to reach and surpass *Healthy People 2020* targets for reducing tobacco use. This can be accomplished through increased tobacco screening and cessation counseling in health care settings (5). Recommended by the US Public Health Service Clinical Practice Guideline for Treating Tobacco Use and Dependence (Guideline), clinician intervention increases the chances of smokers’ quitting (6). The Guideline recommends that clinicians screen for tobacco use, document status, and provide brief treatment using a 5 A’s clinical protocol. The 5 A’s are 1) Asking patients about tobacco use, 2) Advising users to quit, 3) Assessing their willingness to quit, 4) Assisting with their quit attempt, and 5) Arranging for follow-up contact.

Surveys are commonly used to monitor, inform, and evaluate adherence to the clinical practice guideline among clinicians (7). Consistent and effective use of the 5 A’s protocol is low. Clinicians report frequent asking and advising but less frequent assessing, assisting, or arranging (8–12). This case study describes the process for conducting a pre-intervention survey of practices and beliefs regarding treatment of tobacco use among clinicians in Louisiana’s public hospital system.

## Community Context

In 2001, Louisiana’s state legislature addressed the state’s high smoking prevalence (25%) by increasing the excise tax on cigarette sales from 24 cents to 36 cents per pack, ranking Louisiana 49th among the states (13). A dedicated portion (2 cents of the 12-cent increase) of the revenue went toward establishing tobacco control programming for the state, including cessation services for patients in the public hospital system (14). The 10-facility safety-net system, which provides care for residents who are most medically vulnerable, is managed by the Louisiana State University (LSU) Health System. In 2011, the LSU Health System recorded more than 66,000 in-patient admissions and 2.1 million outpatient visits (15). The LSU Health System has 2 distinct institutions operating separate hospitals: the Health Care Services Division (HCSD), which operates 7 hospitals in the southern part of the state; and the LSU Health Sciences Center Shreveport, which operates 3 hospitals in the northern part of the state.

Because of differences in the type of system technology used by each operating institution, the study was conducted only in HCSD hospitals. Of all primary care outpatients in the HCSD system, 65% were women, 53% were African American, 42% were uninsured, and on average, patients were 42 years old, and 29% were current smokers (Y Yi, PhD, written communication, July 2013). The HCSD system also serves as the largest health care training program for physicians, nurses, and allied health students in the state (15).

In 2002, the HCSD, in partnership with the LSU Health Science Center School of Public Health, established the Tobacco Control Initiative (TCI), which used a comprehensive, theory-driven, structured approach to integrate Guideline recommendations. Strategies addressed tobacco use treatment at the system, clinic, and patient levels (16). To monitor and improve provider adherence to Guideline recommendations, the TCI conducts patient surveys to assess their tobacco use, quit attempts, and perceptions of provider treatment (17), and electronic health record (EHR) queries to examine clinician practice patterns. The Table reports the frequencies of 5 A’s performance from a patient survey and EHR query from January through March 2010. However, the TCI had not conducted a system-wide survey of clinicians engaged in treatment of tobacco use to assess their reported adherence to the 5 A’s protocol or to identify belief factors that promote or hinder provision of this evidence-based intervention.

**Table:** Frequencies of 5 A’s Performance From TCI Patient Survey and EHR Query in the LSU Health System Among Primary Care Outpatients, January–March 2010

5 A’s Protocol	Frequency (%)	
	Patient Survey 2010	EHR Query 2010
**Asked** about tobacco use.	740/820 (90.2)	39,424/48,913 (80.6)
**Advised** to quit smoking.	164/192 (85.8)	11,224/11,372 (98.7)
**Assessed** willingness to make a quit attempt.	136/192 (71.7)	11,224/11,372 (98.7)
**Assisted** with selecting or prescribing/recommending a treatment option.	124/164 (76.3)	2,183/3,220 (67.8)
**Arranged** follow-up contact within 1 week or 1 month.	48/164 (31.4)	2,183/2,183 (100.0)

Abbreviations: TCI, Tobacco Control Initiative; EHR, electronic health record; LSU, Louisiana State University.

In 2011, the TCI applied for and was awarded a National Cancer Institute (NCI) Research to Reality (R2R) Mentorship Program mentee position. The R2R mentorship program pairs experienced public health professionals (mentors) with mid-level cancer prevention and control managers (mentees) to build mentees’ capacity to effectively navigate the complexities in which evidence-based intervention occurs. With the mentor’s expertise, and training from the NCI, the TCI mentee planned a year-long cancer prevention and control project relevant to the TCI program to enhance and apply the mentee’s skills and knowledge of evidence-based public health (18). The objectives of the R2R project were to 1) conduct a pre-intervention assessment of tobacco treatment practices and beliefs among clinicians in the HCSD and 2) engage HCSD clinicians to obtain their perspectives on adherence to the Guideline by using an electronic survey.

## Methods

**Partnering with the health care community.** To administer the survey, the TCI leadership identified key partners in the HCSD system based on pre-existing relationships. Partners included the HCSD chief medical officer (CMO) and director of staff development (DoSD). Partners were recruited during HCSD meetings and via e-mail. The TCI sought assistance from the partners at the beginning of study development for approval to conduct the survey, for endorsement by the CMO of correspondence regarding the survey, and for identification by the DoSD of an electronic method to administer the survey. Additionally, the mentorship pair met individually with the CMO during a planned site visit to understand first-hand the organization and context in which the study would be conducted.

**Identifying and adapting an evidence-based clinician survey.** Literature searches were conducted by the mentorship pair to identify existing instruments used to measure clinician practices and beliefs regarding adherence to the Guideline. Authors of published surveys were contacted for permission to use their instruments. Once received, to ensure all questions were relevant and appropriate, the instruments were reviewed for possible adaptation. Changes to the survey were made to reflect the setting (hospital vs clinic) and available cessation resources. The selected questionnaire consisted of 5 sections: 1) practice characteristics, 2) adherence to Guideline recommendations, 3) knowledge, attitudes, and beliefs regarding treatment of tobacco use, 4) current and former tobacco use, and 5) demographic information. Questions regarding clinician 5 A’s practices included the following: “In your hospital/clinic, do you personally ever ask patients if they smoke/use spit or smokeless tobacco?”(responses recorded as yes or no); “How often do you do the following in your hospital/clinic: Advise smokers to stop smoking?; Ask smokers if they are interested in quitting?; Encourage smokers who want to quit to set a quit-date?; Discuss medication options such as nicotine replacement, Chantix (Varenicline) or Zyban (Bupropion SR)?; Refer to the Tobacco Control Initiative (TCI) for treatment?; and Re-evaluate tobacco use at each visit?”(responses were recorded as never, rarely, sometimes, often, or always).

**Developing the survey protocol.** Surveys were scheduled for an 8-week period of administration, beginning in February 2012. The protocol included 1) e-mailing a survey notification letter in week 1 to introduce the upcoming survey, 2) e-mailing an invitation letter in week 2, and 3) e-mailing reminder letters at weeks 3, 5, and 7 to complete the survey. The TCI leadership and mentee drafted correspondence letters regarding the survey for distribution by the CMO. The letters were approved and the project was endorsed by the CMO. A consent form was developed to inform participants of all aspects of the study. Surveys were anonymous and optional, and incentives were not provided to encourage participation. Clinicians could participate in the survey at any time using computers at work or at home. Although no steps were taken to prevent individuals from responding more than once, survey collector settings were set to 1) allow multiple responses per computer as recommended for kiosks or computer laboratories where multiple providers would potentially complete the survey in the hospital, and 2) allow responses to be edited so that respondents could go back to previous pages in the survey and update existing responses until the survey was finished or until they had exited the survey. After the survey was finished, or once respondents exited the survey, respondents were not able to re-enter the survey. On survey completion, respondents were redirected to the HCSD TCI homepage. We did not save Internet Protocol (IP) addresses in the survey results to ensure anonymity. This study was approved by the LSU Health Sciences Center Institutional Review Board.

**Administering the clinician survey.** The TCI and the DoSD worked together to identify a survey delivery method and sampling frame. The team chose the HCSD’s Web-based In-service Learning Management Application (WILMA). WILMA is a provider e-learning management system used to train and track mandatory provider education activities. WILMA was identified as a medium for electronic administration of the survey because 1) WILMA contains a built-in e-mail notification system widely used by each HCSD facility for announcements and lessons assigned to clinicians, 2) clinicians are responsible for checking WILMA on a weekly basis for new information, 3) WILMA allows 1 person to send information out to all clinicians via e-mail and develop statistical reports, and 4) it provides the capability to host a Survey Monkey link. Survey Monkey, an Internet-based tool for conducting Web-based surveys using multiple question formats, offered a cost-effective, self-service surveying solution with built-in privacy and security features. Fifty-two survey items were entered into the Survey Monkey Web interface. The TCI tested the format and configuration of the survey on the Survey Monkey website. All clinicians were sent an invitation and reminder letters via WILMA, which included an embedded link to the survey. Thus, the sampling frame included physicians (primary care, emergency department, and subspecialties) and nurses (nurse practitioners, registered nurses, and licensed practical nurses) with an LSU e-mail address.

## Outcome

Partnering with the hospital system was successful, although the proposed timeline for project completion was not achieved. Identifying and adapting an existing survey, completing and obtaining approval of the institutional review board application, and accommodating facility transitions into partnerships with private health care delivery systems delayed the start of the survey.

**Health care community partnerships.** By partnering with the health care community, the TCI was able to obtain endorsement for the project, access and engage clinicians, and help the system to identify an innovative and efficient use of an existing resource (WILMA).

**Survey identification and adaptation.** Identifying a suitable survey was more time-consuming than expected. Beginning in December 2011, several attempts were made via telephone and e-mail to obtain the text of the first identified survey. However, correspondence with the author during the course of 7 weeks did not result in receipt of the instrument. Conversely, the second identified survey was obtained after 1 request.

**Figure 1:**
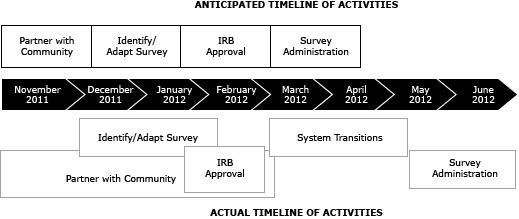
Anticipated and actual timeline of activities. Abbreviation: IRB, institutional review board.

**Protocol for survey distribution.** System-wide layoffs by the HCSD in February 2012 presented another unanticipated delay. Although terminated employees were not present physically, they were not officially removed from the HCSD Department of Human Resources’ (HR) employee management system (which populates WILMA). Beginning survey administration before HR could reconcile its management system would result in issuing a greater number of surveys than the number of clinicians employed, thereby skewing the data. The DoSD suggested that the survey not begin until the hospital’s e-mail system was recalibrated in April 2012. Because of these delays, the survey was not distributed until May 2012. Figure 1 compares the anticipated and actual timeline of events. Additionally, the original protocol included e-mailing a survey notification letter, invitation letter, and 3 reminder letters. Because of complaints received from physicians at 1 facility, the third reminder letter was not e-mailed at the request of the CMO.

**Responses to survey administration*.*** The sample population consisted of 2,519 physicians (including primary care and general practice physicians, obstetricians, gynecologists, emergency medicine physicians, and other specialty physicians) and 1,989 nurses (including registered nurses, licensed practical nurses, and nurse practitioners) in the HCSD system. Among all respondents, 75% were women, 72% were white, ages ranged from 22 to 64 (22–44 [48%] and 45–64 [48%]), and 10% reported current smoking. 

**Figure 2:**
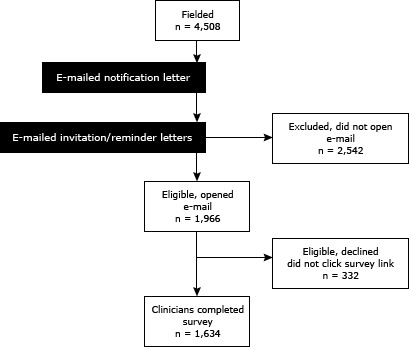
Flow diagram of survey response.

Overall survey response is summarized in Figure 2. Of the 2,519 physicians sent an e-mail containing the invitation letter to participate in the study, 610 (24.2%) opened the e-mail, and 576 (94.4%) opened the letter and clicked the Web link to complete the survey. Of the 1,989 nurses who were sent an e-mail containing the invitation letter, 1,356 (68.2%) opened the e-mail, and 1,058 (78%) opened the letter and clicked the Web link to complete the survey. Using the cooperation rate calculation (7) defined by the American Association of Public Opinion Research (the number of completed surveys [1,634] divided by a denominator that excludes all cases unable to be contacted [1,966]), the response rate was 83.1%. Using the AAPOR’s more conservative calculation (the number of completed surveys [1,634] divided by the total number fielded [4,508]), the response rate was 36.2%. 

**Figure 3:**
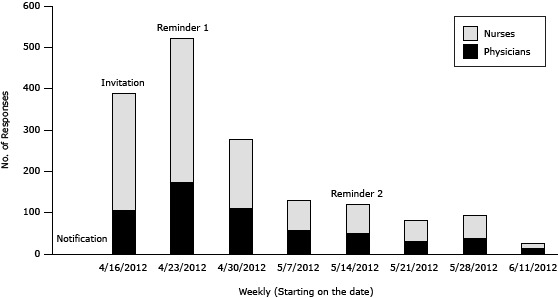
Weekly survey responses among physicians and nurses.

**Figure 3** shows the weekly number of responses among physicians and nurses. For this study, we did not conduct follow-up contact to convert initial nonrespondents into respondents because of the anonymous administration of the survey. We did not save IP addresses or collect identifiable contact information in the survey, as required by the institutional review board. Therefore, we could not follow-up with nonresponders.

**Table:** 

Week by date in 2012	4/16^a^	4/23^b^	4/30	5/7^c^	5/14	5/21^d^	5/28	6/4	6/11
Nurse response	0	283	347	166	73	70	49	57	13
Physician response	0	106	174	110	56	49	31	37	13

^a^ Notification letter sent.

^b^ Invitation letter sent.

^c^ Reminder letter 1 sent.

^d^ Reminder letter 2 sent.

## Interpretation

To effectively engage and support the HCSD community of clinicians in the treatment of tobacco use, it was necessary to conduct an assessment of clinicians (19). Understanding clinician perspectives on Guideline implementation is imperative for improving health care delivery and related policy (20).

This R2R mentorship project was valuable for the mentee, the parent program, and the health care community in which it was conducted. First, the R2R project provided the mentee a foundation in evidence-based public health. The NCI trainings allowed the mentee to gain a greater understanding of how to partner with a hospital community, engage clinicians to assess their practices and beliefs regarding treatment of tobacco use, and collect, analyze, and interpret survey data. Second, the project made a substantive contribution to the TCI parent program. Through the TCI program, Guideline recommendations for treatment of tobacco use were successfully implemented in a public health care system (16). Patient and EHR data are used to assess Guideline adherence; however, provider perspectives had not been obtained. The R2R mentorship project gave the TCI an opportunity to assess clinician practices and beliefs. This information will guide the development of interventions to improve clinician adherence to the Guideline.

There was also an unforeseen benefit of the R2R project. Through the project, an existing resource was identified for conducting research in the HCSD system. A review of health care provider surveys published between 2000 and 2010 found that the number of provider surveys (primarily mailed surveys) with a 60% response rate (minimum requirement) had been declining (7). Clinician e-mails can increase Internet and e-mail data collection methods (20). Using e-mail for initial contact and the Internet for survey data collection, our study achieved a 94% cooperation rate among physicians and a 78% cooperation rate among nurses. E-mail/Internet administration appears to have resulted in higher cooperation rates, and these delivery methods may be more efficient in gathering health-care provider input. WILMA provided a single portal to access all clinicians in the HCSD system via e-mail. Contact information was current and specific to the population of interest. The R2R project demonstrated that other electronic-based methods can become a part of the research infrastructure.

To those who intend to conduct similar research in the hospital community, authors stress the importance of involving all stakeholders early in the process, using existing infrastructure to collect information, and including enough time in the implementation schedule to accommodate protocol changes. Partnering with the hospital community was a viable approach to determine clinician practices and beliefs about the treatment of tobacco use and adherence to the Guideline.

## Figures and Tables

**Table:** 

**1. The activity supported the learning objectives.**				
**Strongly Disagree**				**Strongly Agree**
1	2	3	4	5
**2. The material was organized clearly for learning to occur.**				
**Strongly Disagree**				**Strongly Agree**
1	2	3	4	5
**3. The content learned from this activity will impact my practice.**				
**Strongly Disagree**				**Strongly Agree**
1	2	3	4	5
**4. The activity was presented objectively and free of commercial bias.**				
**Strongly Disagree**				**Strongly Agree**
1	2	3	4	5

## Post-Test Information

To obtain credit, you should first read the journal article. After reading the article, you should be able to answer the following, related, multiple-choice questions. To complete the questions (with a minimum 75% passing score) and earn continuing medical education (CME) credit, please go to http://www.medscape.org/journal/pcd. Credit cannot be obtained for tests completed on paper, although you may use the worksheet below to keep a record of your answers. You must be a registered user on Medscape.org. If you are not registered on Medscape.org, please click on the "Register" link on the right hand side of the website to register. Only one answer is correct for each question. Once you successfully answer all post-test questions you will be able to view and/or print your certificate. For questions regarding the content of this activity, contact the accredited provider, CME@medscape.net. For technical assistance, contact CME@webmd.net. American Medical Association's Physician's Recognition Award (AMA PRA) credits are accepted in the US as evidence of participation in CME activities. For further information on this award, please refer to http://www.ama-assn.org/ama/pub/about-ama/awards/ama-physicians-recognition-award.page. The AMA has determined that physicians not licensed in the US who participate in this CME activity are eligible for **AMA PRA Category 1 Credits**™. Through agreements that the AMA has made with agencies in some countries, AMA PRA credit may be acceptable as evidence of participation in CME activities. If you are not licensed in the US, please complete the questions online, print the AMA PRA CME credit certificate and present it to your national medical association for review.

## Post-Test Questions

### Article Title: Partnering With Health Care Systems to Assess Tobacco Treatment Practices and Beliefs Among Clinicians: Evaluating the Practice

**CME Questions**

1. Which of the following statements regarding sociodemographic variables associated smoking status among residents of Louisiana is most accurate?
  - Not having health insurance is associated with a higher rate of smoking
  - Gender has no effect on the rate of smoking
  - The prevalence of smoking is increasing fastest among college graduates
  - Income has no effect on the rate of smoking
2. Clinicians perform ***best*** in which of the "5 A’s" in clinical practice?
  - Asking about tobacco use
  - Assessing willingness to quit
  - Assisting with a quit attempt
  - Arranging for follow-up
3. Which of the following statements regarding the response to the email invitation for participation in the current study is ***most*** accurate?
  - Most physicians opened the email letter
  - Most physicians who opened the letter activated the embedded Web link
  - A higher proportion of physicians opened the letter compared with nurses
  - Few health personnel who opened the letter actually completed the survey
4. Which of the following characteristics describes survey respondents in the current study?
  - Usually men
  - Usually white
  - Nearly 90% younger than 45 years
  - Collective rate of smoking less than 1%

**Evaluation**
